# Mechanisms driving the functional maturation of the developing mammalian auditory pathway

**DOI:** 10.1016/bs.ctdb.2025.03.005

**Published:** 2025-04-16

**Authors:** Federico Ceriani, Katherine C. Wood, Stuart L. Johnson, Corné J. Kros, Walter Marcotti

**Affiliations:** a School of Biosciences, University of Sheffield, Sheffield, United Kingdom; b Neuroscience Institute, University of Sheffield, Sheffield United Kingdom; c Sussex Neuroscience, School of Life Sciences, University of Sussex, Falmer, Brighton, United Kingdom

## Abstract

The accurate representation of sound in the central auditory pathway of mammals depends on the cochlea, the peripheral sensory organ, which is optimised to detect acoustic signals with unparalleled temporal precision. Beyond its role in converting acoustic stimuli into electrical signals, the cochlea also plays a key role in shaping the maturation of the auditory pathway during pre-hearing stages. This process is essential for creating the tonotopic maps used to identify a broad range of sound frequencies. To achieve this extraordinary task, the sensory hair cells and supporting cells of the pre-hearing cochlear sensory epithelium generate spontaneous, sensory-independent Ca^2+^ signals that propagate along the ascending auditory pathway. Here we review the current understanding of how the different Ca^2+^ signals are generated within the developing cochlea, how they interact to regulate the activation of the auditory afferent fibres, and how they ultimately contribute to the establishment of a mature auditory system pathway. Remarkably, a partial regression to an immature developmental stage occurs in the ageing cochlea, correlated with age-related hearing loss. Increasing our understanding of how the cochlear epithelium changes during all stage of life will inform future therapies for preventing and to reverse hearing loss.

## Abbreviations

ATP: Adenosine triphosphate
ABR: Auditory brainstem response
AC: Auditory cortex
AN: Auditory nerve
BM: Basilar membrane
CNS: Central nervous system
Cb: Cerebellum
CN: Cochlear nucleus
Cx: Connexin channel
DAG: Diacylglycerol
E: Embryonic day
EP: Endocochlear potential
ER: Endoplasmic reticulum
ERK1/2: Extracellularly regulated kinases 1 and 2
GER: Greater epithelial ridge
IC: Inferior colliculus
IP_3_: Inositol 1,4,5-trisphosphate
IHC: Inner hair cell
BK or K,f: Large conductance Ca^2+^ activated K^+^ channels
LOC: Lateral olivocochlear efferent
LSO: Lateral superior olive
LER: Lesser epithelial ridge
MET: Mechano-electrical transducer current
MGB: Medial geniculate nucleus
MOC: Medial olivocochlear efferent
MNTB: Medial nucleus of the trapezoid body
nAChRs: Nicotinic acetylcholine receptors
OHC: Outer hair cell
PLC: Phospholipase C
P: Postnatal day
P2: Purinergic receptor
SK2: Small conductance Ca^2+^ activated K^+^ channels
SGN: Spiral ganglion neuron
SC: Superior colliculus
SOC: Superior olivary complex
SC: Supporting cell
TM: Tectorial membrane
TMEM16a: Transmembrane protein 16 A

## 1 Introduction

**Fig. 1:**
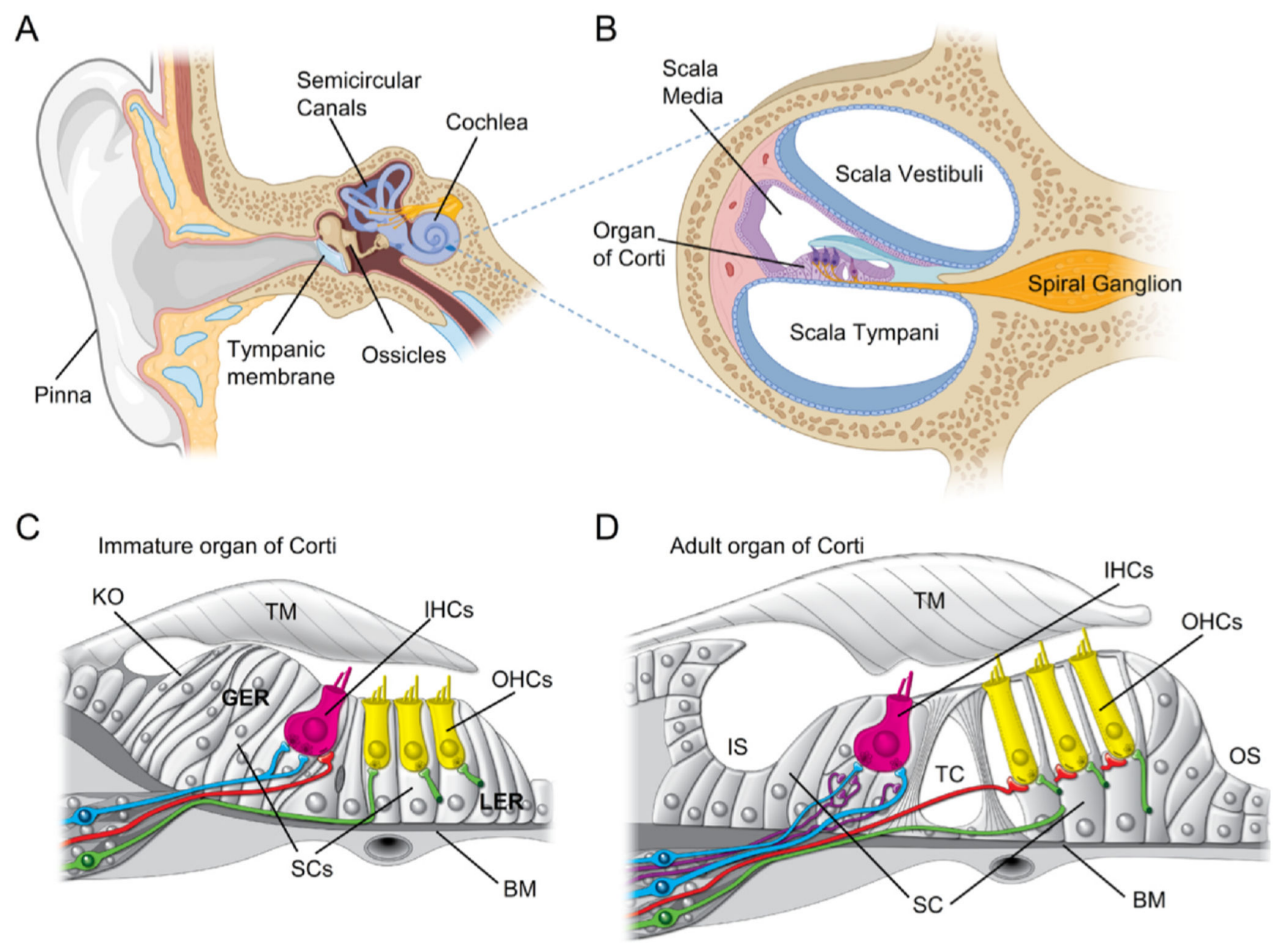
The structure of the ear. (A) Schematic diagram of the human ear showing the external ear consisting of the pinna and ear canal, the middle ear consisting of the tympanic membrane and ossicles, and the inner ear consisting of the vestibular system replete with three semicircular canals, utricle, saccule and the coiled cochlea. (B) Zoomed in cross section through one turn of the cochlea showing the three chambers that form the cochlea, which are the scala vestibuli, the scala media and the scala tympani. The organ of Corti sits at the bottom of the scala media. The spiral ganglion contains the cell bodies of the afferent neurons that innervate the organ of Corti. (C,D) Schematic drawings of the organ of Corti from an immature prehearing cochlea, (C, during the first postnatal week in the mouse) and a morphologically mature cochlea, (D, after the onset of hearing at P12 in the mouse). The images show the single row of inner hair cells (IHC) and three rows of outer hair cells (OHCs). Greater epithelial ridge (GER); Lesser epithelial ridge (LER). Kolliker’s organ (KO) is present in the immature cochlea on the medial side of the IHCs. The tectorial membrane (TM) and the basilar membrane (BM) are shown. A type-I spiral ganglion neuron (light blue), a type-II spiral ganglion neuron (green) and a medial olivocochlear (MOC) efferent fibre (red) are shown innervating the immature cochlea. Note that in the immature cochlea the type-I fibre is branched and the MOC efferent innervates the IHC. The truncated type-II fibres (green) below the OHCs (yellow) indicate that they project from a more apical region of the organ of Corti. In addition to hair cells, the organ of Corti also has several types of supporting cells (SCs) (see Kelley, 2022). In the adult cochlea, medial to the organ of Corti is the inner sulcus (IS), a region that has formed from Kolliker’s organ, and the tunnel of Corti (TC), flanked by supporting cells called pillar cells (PC), has opened up. The outer sulcus (OS) represents the lateral third of the organ of Corti (*i.e*., towards the stria vascularis). In the adult cochlea, the type-I fibres are not branched, MOC efferents (red) innervate the OHCs and lateral olivocochlear (LOC) efferent fibres (purple) innervate the afferent axons from the type-I SGNs just below the IHCs. Diagrams in panels A and B were created with BioRender.

The auditory system in mammals is finely tuned to detect sound with remarkable precision and sensitivity. In humans, hearing spans an extensive dynamic range, enabling the detection of sounds across a trillion-fold variation in acoustic power, from the faintest sound of a pin dropping to the powerful roar of a jet engine. Sound enters the ear and travels along the ear canal, then strikes the ear drum (tympanic membrane; Fig. 1A). Vibrations of the ear drum are transmitted to the oval window of the cochlea via the ossicles in the middle ear. The cochlea is a spiral-shaped, fluid-filled structure in the inner ear that consists of three parallel chambers, the scala vestibuli, scala media, and scala tympani (Fig. 1A and B).

Within the scala media lies the organ of Corti, the sensory organ of the cochlea that contains specialised mechanosensory hair cells. Sound-induced vibrations of the oval window create traveling waves along the basilar membrane that is found below the organ of Corti. The location of maximal vibration along this membrane varies with the sound’s frequency, allowing the cochlea to resolve complex incoming sounds into their individual frequency components. This organization, known as tonotopic organization, is preserved throughout the entire ascending auditory pathway, from the cochlea to the auditory cortex. The frequency selectivity of the basilar membrane allows us to discriminate individual tones separated by just 0.2 % in frequency, equivalent to one-thirtieth of the interval between consecutive piano keys (Hudspeth & Martin, 2024; Marin et al., 2022).

Like in other sensory systems (Kirkby et al., 2013), the high sensitivity of the auditory system relies on the precise formation of sensory circuits during development (Kandler et al., 2009). While the initial wiring of these circuits depends on axon guidance molecules (Fekete & Campero, 2007; Huberman et al., 2008), their mature structure and function are shaped by spontaneous patterned electrical activity that occurs before external sensory input is detected (*e.g*., auditory: Clause et al., 2014; Kersbergen et al., 2022; Müller et al., 2019; visual: Katz & Shatz, 1996; Tiriac & Feller, 2022). In the developing auditory system, periodic bursts of spontaneous action potentials have been recorded from neurons throughout the central auditory pathway (Babola et al., 2018; Lippe, 1994; Sonntag et al., 2009; Tritsch et al., 2010). Several lines of evidence indicate that this central activity originates in the cochlea since ablation of the sensory epithelium eliminates electrical activity in the auditory centres and disrupts their normal maturation (Lippe, 1994; Mostafapour et al., 2000; Tierney et al., 1997). Importantly, the firing activity travelling along the auditory pathway is initially triggered by the release of glutamate from the sensory inner hair cells (IHCs) (Glowatzki & Fuchs, 2002; Johnson et al., 2019), a process driven by the interplay of multiple mechanisms within the cochlea. This cochlear electrical activity not only regulates the functional maturation of the auditory pathway but also instructs the normal biophysical and morphological maturation of the IHCs themselves (Carlton et al., 2023; Johnson et al., 2007; Johnson et al., 2013).

In this chapter, we provide an overview of the current understanding of the molecular and cellular mechanisms involved in the functional maturation of the mammalian auditory system. We focus on the origin and modulation of the spontaneous Ca^2+^-dependent electrical activity in the cochlea and its influence on the developing auditory pathway.

## 2 Functional maturation of the cochlear sensory epithelium

The adult mammalian cochlea is a highly organised sensory epithelium consisting of two types of polarised sensory hair cells (see also Kindt and Tarchini **Chapter 3** this volume for further details) and an array of non-sensory supporting cells that run the entire length of the cochlea (Fig. 1C and D). IHCs form a single row and act as the primary sensory receptors. They are responsible for relaying all the acoustic information, encoded in the sound waves entering the cochlea, onto the auditory type I afferent fibres (reviewed by: Johnson et al., 2019). Outer hair cells (OHCs) are arranged in three rows and their role is to enhance the sensitivity and frequency selectivity of the cochlea. This is achieved through the voltage-dependent electromotility of their cell bodies (reviewed by: Fettiplace & Hackney, 2006), mediated by the motor protein prestin (Zheng et al., 2000). The cochlea is tonotopically organised, with hair cells at the base being tuned to high sound frequencies and those at the apex tuned to low frequencies. This organisation is primarily determined by the mechanical properties of the basilar membrane that lies beneath the organ of Corti (Fettiplace & Hackney, 2006).

In the mature cochlea, sound-induced deflection of the stereociliary bundle on the apical surface of the hair cells open mechano-electrical transducer channels (see also **Chapter 2** for further details), generating a depolarizing inward current (reviewed by: Fettiplace & Kim, 2014; Marcotti, 2012). In IHCs, this depolarization creates a receptor potential that triggers the activation of Ca_v_1.3 Ca^2+^ channels and the subsequent Ca^2+^-dependent fusion of glutamate-containing vesicles at specialised ribbon synapses. These synapses form a one-to-one connection with the type I afferent terminals (Johnson et al., 2019). The activity of type I afferents is further modulated by the efferent system, which forms axo-dendritic synapses with the afferent terminals (reviewed by: Fuchs & Lauer, 2019). In contrast, the receptor potential in OHCs drives electromotility, which forms the basis of cochlear amplification (reviewed by: Fettiplace & Hackney, 2006). Unlike IHCs, OHC activity is directly modulated by the cholinergic efferent system synapsing onto OHCs (Fuchs & Lauer, 2019).

In addition to sensory hair cells, the organ of Corti also contains a diverse population of non-sensory supporting cells, each with distinct morphological and functional characteristics (reviewed by: Raphael & Altschuler, 2003). Unlike hair cells, which are confined to the luminal surface of the organ of Corti, supporting cells span the full thickness of the epithelium, extending from the underlying basal lamina to the lumen of scala media (Wan et al., 2013) (Fig. 1C and D). This structural arrangement allows supporting cells to interact with various cellular and extracellular compartments, reflecting their multifunctional roles. Supporting cells share functional similarities to glial cells in the central nervous system (CNS) (Monzack & Cunningham, 2013), highlighting their importance in maintaining cellular homeostasis and supporting sensory cell function. These roles include the uptake and spatial buffering of potassium ions (Hibino & Kurachi, 2006; Jagger & Forge, 2006; Kofuji & Newman, 2004; Zdebik et al., 2009), the clearance of synaptically released glutamate (Glowatzki et al., 2006) and the release of trophic factors that support neuronal survival and synapse maintenance (Zilberstein et al., 2012; Zuccotti et al., 2012).

The development of a fully functional auditory system is a highly intricate process, governed by both genetic and activity-dependent factors. Disruptions in these regulatory mechanisms can lead to hearing impairments.

### 2.1 Key molecules involved in mammalian cochlear development

The early development of the mammalian cochlea is primarily guided by intrinsic genetic programmes (see also Nelson, Hosamani and Groves **Chapter 1** this volume for further details). In brief, the mouse cochlear duct begins to emerge and extend from around embryonic day 11 (E11), a process that requires *sonic hedgehog* (*Shh*) signalling. At this early stage, the developing cochlear duct, which will later become the scala media containing the sensory epithelium, is already divided into five distinct regions, each characterised by the expression of specific genes: *Otx2* in the prospective Reissner’s membrane, *Lmo4* in the stria vascularis, *Bmp4* in the lesser epithelial ridge (LER), *Sox2* in the pro-sensory domain and *Fibroblast growth factor 10* (*Fgf10*) in Kölliker’s organ. Notably, genes such as *Sox2, Fgf10* and *Lunatic fringe* (*Lfng*) are also expressed in overlapping patterns between the pro-sensory domain and Kölliker’s organ (reviewed by: Driver & Kelley, 2020; Michalski & Petit, 2019). The cochlear duct continues to extend until about postnatal day 3 (P3) (McKenzie et al., 2004). During this period, most cells in the duct continue to proliferate, except for those in the pro-sensory population, which will give rise to hair cells and supporting cells. These pro-sensory cells exit the cell cycle in a tonotopic gradient, beginning at the apex at E12 and progressing towards the base by E14 (Chen & Segil, 1999; Driver & Kelley, 2020). Following terminal mitosis, pro-sensory cells undergo functional differentiation that progresses in the opposite direction, starting at the base immediately after E14 and progressing towards the apex. This process of differentiation is governed by gradients in the expression of *Atoh1* and *Shh* (Driver & Kelley, 2020).

In addition to tonotopic differentiation, cochlear development is also regulated along the medial-lateral axis, which is essential for establishing the mosaic arrangement of hair cells and supporting cells (Chen et al., 2002; Driver & Kelley, 2020; McKenzie et al., 2004). This process specifies the supporting cells of Kölliker’s organ (which eventually become the inner sulcus cells in the adult), and a single row of IHCs, pillar cells, Deiters’ cells, three rows of OHCs, and outer sulcus cells (Fig. 1C and D). Opposing gradients of *Bone morphogenetic protein 4* (*Bmp4*), *Fgfs* and *Wnt* have been shown to play a key role in defining this medial-lateral axis (Driver & Kelley, 2020; Ellis et al., 2019; Geng et al., 2016). The complex pattern of hair cells and supporting cells is mediated by lateral inhibition involving *Notch* signalling. *Atoh1* expression in developing hair cells activates *Notch* signalling in adjacent cells, leading to the upregulation of *Hes1* and *Hes5*. These factors in turn suppress *Atoh1*, preventing neighbouring cells from becoming hair cells (Driver & Kelley, 2020; Lanford et al., 2000; Zheng et al., 2000; Zine et al., 2001).

**Fig. 2:**
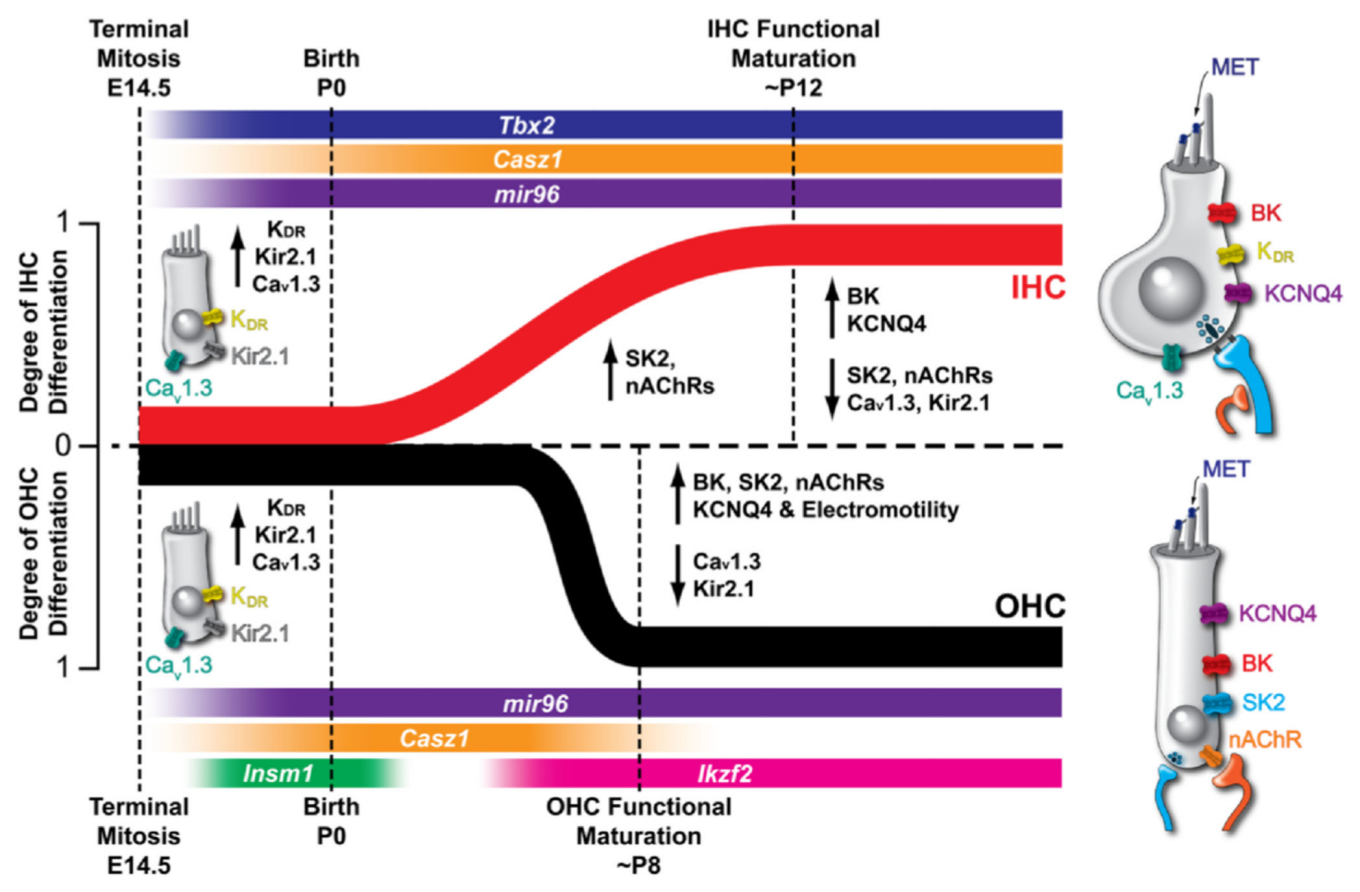
Timeline showing the main physiological events in mouse hair cell development. Schematic diagram showing the degree of functional differentiation in mouse cochlear hair cells as a function of time, where 1 indicates fully differentiated IHCs and OHCs and 0 represents biophysically indistinguishable hair cells. The normal onset (↑) and offset or downregulation (↓) of expression of different types of membrane channels and other membrane proteins in IHCs (top half, red) and OHCs (bottom half, black) is indicated by the arrows. The expression of key genes involved in the functional differentiation of IHCs (*Tbx2*, dark blue; *Casz1*, orange; *miR-96*, purple) and OHCs (*Insm1*: green; *Ikzf2*; pink; *Casz1*, orange; *miR-96*, purple) are indicated. Before birth, hair cells are characterized by similar immature characteristics such as the inwardly rectifying K^+^ channels Kir2.1 carrying *I*_K1_, the outwardly delayed-rectifying K^+^ channels (K_DR_) and the Ca^2+^ channels (Ca_V_1.3). Immature IHCs also express the small-conductance Ca^2+^-activated K^+^ channels (SK2) and α9α10 nicotinic acetylcholine receptors (nAChRs). These channels are also depicted in the cell membrane of the hair cells (right panels). Note that hair cells increase in size with development and only become morphologically diverse at mature stages. The mechano-electrical transducer current (MET) current appears at or just after birth in both IHCs and OHCs. Adult-type currents initially appear at P12 in IHCs, and are the rapidly activating large-conductance Ca^2+^-activated K^+^ channels (BK), and the negatively activating K^+^ KCNQ4 channels. Adult features appear at around P8 in OHCs, including electromotility, the large conductance Ca^2+^-activated K^+^ channels (BK), SK2, nAChRs and KCNQ4 channels. *Modified from* Kuhn et al. (2011).

Once hair cell fate is determined, their differentiation into OHCs and IHCs is established embryonically when they begin to express K^+^ and Ca^2+^ channels (Fig. 2). This process is determined by different transcriptional regulators: INSM1 is required to consolidate OHC fate (Wiwatpanit et al., 2018), whereas TBX2 is required for IHC identity (Bi et al., 2022; García-Añoveros et al., 2022; Kaiser et al., 2022) (Fig. 2). Moreover, a recent study has shown that CASZ1 is essential for IHC fate stabilization and OHC survival at postnatal ages (Sun et al., 2025) (Fig. 2). However, the biophysical and morphological differentiation of IHCs and OHCs occurs at around birth in mice, and this also depends on the expression of the non-coding microRNA 96 (*miR-96*, Kuhn et al., 2011) that is expressed from embryonic stages onwards (Kuhn et al., 2011; Lumayag et al., 2013; Sacheli et al., 2009; Weston et al., 2006) (Fig. 2). *miR-96* regulates several downstream developmental genes including *Tmc1, Ptprq* and *Gfi1* (Lewis et al., 2009). Mutations in *miR-96* result in a coherent developmental arrest, preventing the normal biophysical and full morphological maturation of IHCs and OHCs (Fig. 2) (Kuhn et al., 2011) and leads to non-syndromic progressive hearing loss in humans and mice (Lewis et al., 2009; Mencía et al., 2009). OHCs are the first to initiate their functional maturation, beginning from around P6-P8 in mice with the acquisition of electromotility (Abe et al., 2007; Marcotti & Kros, 1999) driven by the motor-protein prestin (Zheng et al., 2000). The postnatal expression of prestin requires Helios, a transcriptional regulator encoded by *Ikzf2* (Chessum et al., 2018). As OHCs mature around P8, their basolateral membrane also undergoes substantial change, including the expression of KCNQ4 channels (Kubisch et al., 1999; Marcotti & Kros, 1999) and the channels sustaining inhibitory modulation by the efferent synapses (SK2 and nAChRs) (Elgoyhen et al., 2001; Marcotti et al., 2004; Oliver et al., 2000) (Fig. 2). In contrast to OHCs, the functional maturation of IHCs occurs later, around P10-P12 (Kros et al., 1998) (Fig. 2), coinciding with the onset of hearing in most altricial rodents (Mikaelian & Ruben, 1965; Shnerson et al., 1982). While it remains unclear whether IHC functional maturation requires any additional transcription factors, this process is associated with the upregulation of KCNQ4 and BK channels (Kros et al., 1998; Marcotti et al., 2003a) and down-regulation of SK2 channels and α9α10nAChRs (Fig. 2). This downregulation aligns with the loss of axo-somatic efferent contact, which occurs at the same time (Glowatzki & Fuchs, 2000; Katz et al., 2004; Marcotti et al., 2004; Simmons et al., 1996).

The genetic programmes that govern cochlear development and organisation are not solely responsible for determining its final structure and function. Instead, genetic programmes are influenced and guided by spontaneous activity generated by immature hair cells before the onset of sensory-driven function (Friauf & Lohmann, 1999). This spontaneous activity plays a vital role in fine-tuning the regulation of genetic programmes at the cellular level, coordinating the activity among adjacent cells, and shaping cellular and neural circuit function before it becomes fully operational. This means that the developmental processes are not strictly genetically predetermined but can be refined up to their final configuration.

## 3 Calcium signals in the developing hair cells

**Fig. 3:**
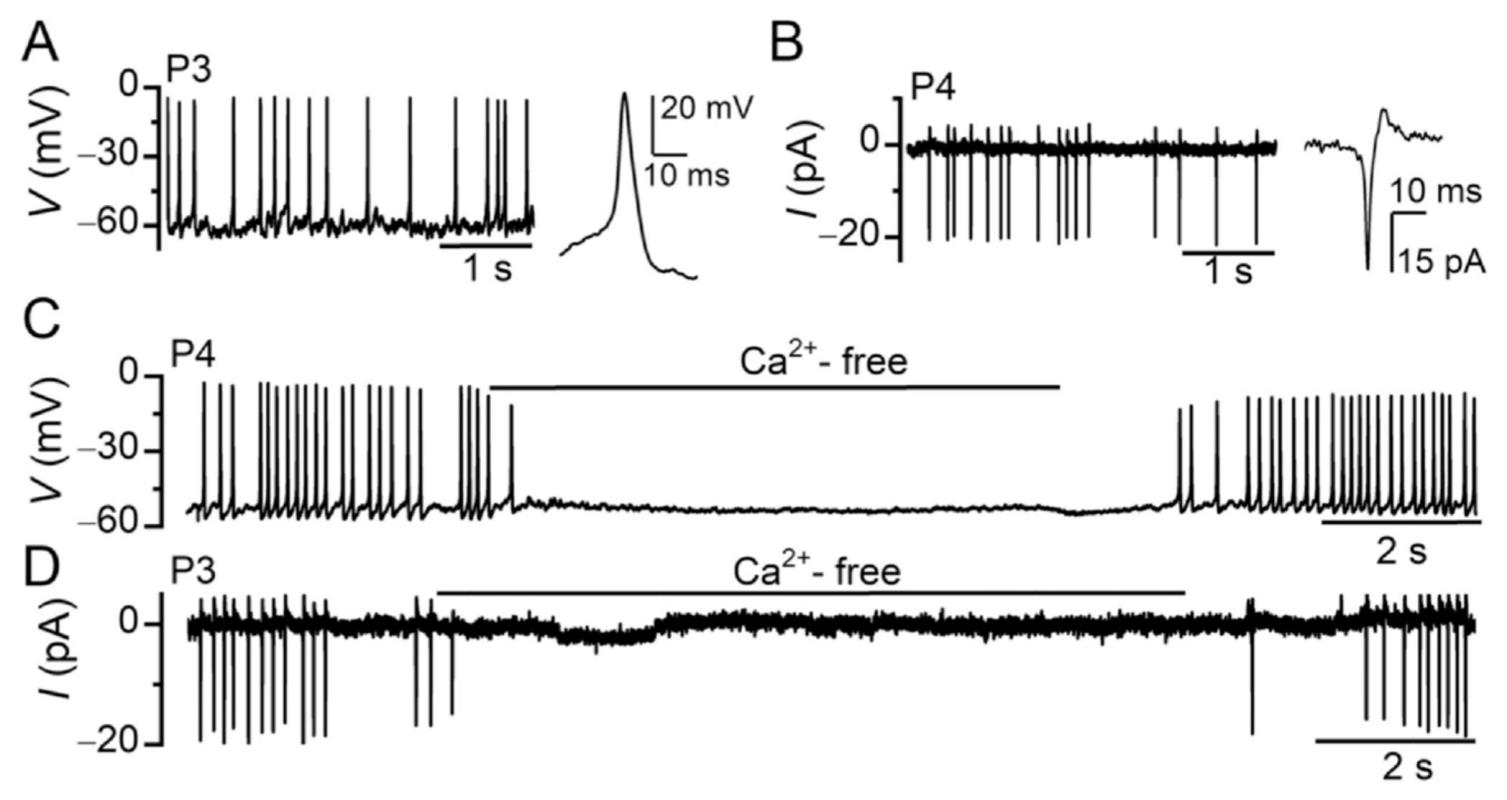
Calcium-dependent action potentials in developing IHCs. (A, B) Action potentials recorded using whole-cell current clamp (A) and cell-attached voltage clamp. (B) The recordings on the right of each panel show a single action potential on an expanded time scale. (C, D) Action potentials in pre-hearing IHCs recorded using whole-cell current clamp (C) and cell-attached voltage clamp (D) are abolished in the absence of extracellular Ca^2+^ (Ca^2+^-free solution). Image from Johnson et al. (2011).

Before the onset of hearing at around P12, mouse IHCs generate spontaneous or evoked action potentials when maintained in *ex vivo* cochlear explants (Beutner & Moser, 2001; Kros et al., 1998; Marcotti et al., 2003a; Marcotti et al., 2003b; Sendin et al., 2014) (Fig. 3). These action potentials are primarily driven by the interplay between an inward Ca^2+^ current through L-type Ca^2+^ channels (Ca_V_1.3), which account for around 90 % of the total Ca^2+^ current in IHCs (Brandt et al., 2003; Platzer et al., 2000), and a delayed outwardly rectifying K^+^ current, which allows sustained K^+^ efflux with a delay following membrane depolarization (Kros et al., 1998; Marcotti et al., 2003b). The removal of Ca^2+^ from the extracellular solution completely and reversibly abolishes the action potentials in IHCs, which can be recorded using either whole-cell current clamp (Fig. 3A and C) or cell-attached voltage clamp (Fig. 3B and D) patch-clamp configurations. Although IHCs located in the apical coil of cochlear explants exhibit a small Ca^2+^ current as early as E16.5 (Marcotti et al., 2003b), Ca^2+^-dependent action potentials first appear one day later (Marcotti et al., 2003a). During embryonic stages, these action potentials are small and slow, but gradually become larger and faster after birth (Marcotti et al., 2003a; Marcotti et al. 2003b; Marcotti et al., 2004). Up to approximately P6, *ex vivo* IHCs generate spontaneous action potentials when electrophysiological recordings are performed using near-physiological recording conditions, including a temperature of 35–37 °C and a perilymphlike extracellular solution (1.3 mM Ca^2+^ and 5.8 mM K^+^) (Johnson et al., 2011). From around P7 up to the onset of hearing, action potentials can only be elicited through IHC depolarization. The disappearance of spontaneous Ca^2+^ action potentials during the second postnatal week is driven by a progressive hyperpolarization of the IHC resting membrane potential to approximately −75 mV (Johnson et al., 2011; Marcotti et al., 1999; Marcotti et al., 2003a), a value more negative than the Ca^2+^ channel activation threshold (about −65 mV: Zampini et al., 2010).

As the IHCs acquire the biophysical characteristics required to elicit action potentials, their postsynaptic afferent fibres, which enter the sensory epithelium before hair-cell differentiation, begin forming axo-somatic contacts with IHCs just before birth (from around E18: Huang et al., 2007; Pujol et al., 1998). At the same time, auditory afferent neurons start expressing glutamate receptors (Luo, Brumm, & Ryan, 1995; Puyal et al., 2002) and the post-synaptic densities in the afferent terminals become colocalised with IHC ribbon synapses (Sobkowicz et al., 1982; Sobkowicz, 1992). Despite the small Ca^2+^ current in the IHCs and the immaturity of the afferent synapses at this stage, individual action potentials in IHCs are already able to trigger the Ca^2+^-dependent fusion of glutamate-containing vesicles with the presynaptic plasma membrane (Johnson et al., 2005; Marcotti et al., 2003a), leading to action potential generation in the afferent neurons as early as P0 (Tritsch & Bergles, 2010). This early electrical activity is unlikely to be limited to the cochlea and could be transmitted to the developing CNS since evidence suggests that functional synapses are already established in the brainstem at birth (Hoffpauir et al., 2006), and neurons in the superior olivary complex can fire action potentials already during embryonic stages (Kandler & Friauf, 1995).

### 3.1 Modulation of the IHC Ca^2+^-dependent firing activity

Several studies have shown that the frequency and pattern of action potential activity in pre-hearing IHCs from cochlear explants can be modulated by intrinsic and extrinsic mechanisms. This diversity in firing modulation seems to be aimed at regulating not only the maturation of the central auditory pathway, but also that of the IHCs themselves. Previous work in other systems has shown that early spontaneous electrical activity is essential for regulating a variety of cellular responses, including the remodelling of synaptic connections (Zhang & Poo, 2001) and ion channel expression (Moody & Bosma, 2005). In IHCs, mechanisms identified that influence action potential activity include intrinsic factors, such as basolateral membrane and mechano-electrical transducer channels, as well as extrinsic influences such as ATP release from the surrounding supporting cells, and activity from the efferent cholinergic system descending from the brainstem.

#### 3.1.1 Sodium channels

**Fig. 4:**
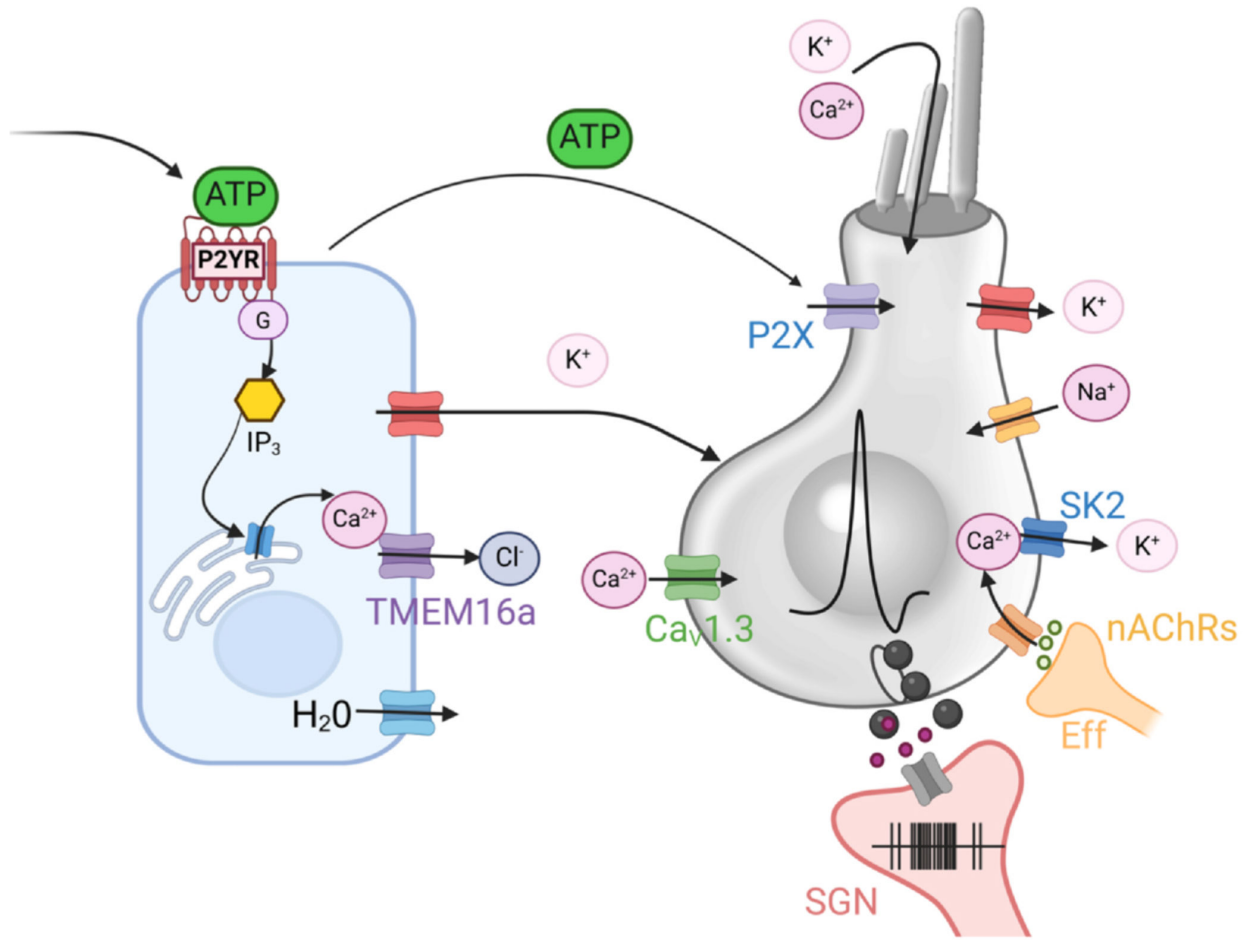
Diagram showing the modulation of electrical activity in developing IHCs. Molecular components in both the supporting cells (left) and IHCs (right) involved in the key mechanisms regulating the action potential activity in developing IHCs. Ca_V_1.3: voltage-gated L-type Ca^2+^; α9α10: nicotinic acetylcholine receptor; SK2: small conductance Ca^2+^-activated potassium channel; IP_3_: Inositol 1,4,5-trisphosphate; TMEM16A: transmembrane protein 16A (also known as Anoctamin-1, ANO1): Ca^2+^-activated Cl^−^ channels; P2YR: G-coupled metabotropic purinergic receptors, K^+^ and Ca^2+^ enter the stereocilia via the mechano-electrical transducer channels; SGN: spiral ganglion neuron (afferent nerve fibre); Eff: efferent nerve fibre. For the mechanisms illustrated see main text. Image was created with BioRender.

In addition to the Ca^2+^ current, some pre-hearing IHCs express a TTX-sensitive Na^+^ conductance (Fig. 4) (Marcotti et al., 2003b; Oliver et al., 1997), which is essential for triggering action potentials in most neurons. In IHCs, the Na^+^ current is not required to trigger an AP, but it directly influences their frequency by shortening the time to reach threshold. Currently, the physiological role of this additional inward current is unclear, especially considering that it is not expressed in all IHCs (Marcotti et al., 2003b).

#### 3.1.2 Small conductance Ca^2+^-activated & inward rectifier K^+^ channels

During pre-hearing stages of development IHCs transiently express small conductance Ca^2+^-activated K^+^ (SK2) channels (Fig. 4) (Glowatzki & Fuchs, 2000; Katz et al., 2004; Marcotti et al., 2004). The size of the SK2 current reaches its peak during the second postnatal week, playing a key role in regulating the frequency and duration of the spontaneous action potentials in IHCs. Studies have shown that either knockout or over-expression of SK2 channels disrupt the temporal pattern of spontaneous action potentials in IHCs (Johnson et al., 2007; Johnson et al., 2013). This disruption impairs the functional maturation of Ca^2+^-induced exocytosis at IHC ribbon synapses, although it does not affect general IHC development. The influence of IHC firing activity on synaptic maturation is confined to a narrow time window during the second postnatal week, just before the onset of hearing (Johnson et al., 2007; Johnson et al., 2013). The existence of this “critical period” is further supported by evidence indicating that the absence of spontaneous Ca^2+^ action potentials in IHCs during the second postnatal week leads to the upregulation of the genetic pathways involved in the maintenance of cytoskeletal homeostasis, which adversely affects the normal morphological and functional maturation of the stereociliary bundle (Carlton et al., 2024).

#### 3.1.3 Mechano-electrical transducer channels

As mentioned above, IHCs in cochlear explants lose the ability to generate spontaneous, but not evoked, action potentials during the second postnatal week. This is attributed to the progressive hyperpolarization of their resting membrane potential. However, when the stereociliary bundles of the IHCs are exposed to a solution with a near endolymphatic Ca^2+^ concentration, the resulting increase in depolarizing mechano-electrical transducer (MET) current flowing into the IHCs (Fig. 4) counteracts the membrane hyperpolarization, thereby restoring spontaneous action potential activity (Johnson et al., 2012). These findings suggest that, *in vivo*, spontaneous action potentials are likely to occur throughout pre-hearing stages. Additional studies have also shown that disrupting the MET current during IHC development, which is likely to disrupt their spontaneous action potential firing, prevents the functional maturation of the sensory epithelium. For example, mice lacking or carrying mutations in key stereociliary bundle proteins essential for mechano-electrical transduction, such as TMC1 (*Deafness* mouse: Marcotti et al., 2006), unconventional MYO6 (*Snell’s waltzer* mouse: Marcotti et al., 2006) and EPS8 (Zampini et al., 2011), fail to upregulate the basolateral membrane channels characteristics of mature IHCs (*I*_*K*,*n*_ and *I*_*K*,*f*_), and exhibit impaired specification of type I afferent neurons into subclasses (Sun et al., 2018). These observations further support the existence of a critical period in IHC development, during which Ca^2+^ activity plays a pivotal role in regulating the final stages of their functional maturation.

#### 3.1.4 ATP released by supporting cells

Before the onset of hearing, supporting cells surrounding IHCs spontaneously release ATP into the extracellular space (Tritsch et al., 2007). ATP has been shown to induce Ca^2+^-dependent AP activity in IHCs through both direct and indirect mechanisms (Fig. 4). Directly, ATP activates P2X receptors expressed in the IHC membrane (Tritsch et al., 2007), while indirectly it triggers a complex signalling cascade leading to the propagation of Ca^2+^ signals as regenerative intercellular Ca^2+^ waves across neighbouring supporting cells (Wang et al., 2015). These Ca^2+^ waves ultimately lead to the efflux of K^+^ into the intercellular space following the opening of Ca^2+^-activated Cl^-^ channels TMEM16A (Fig. 4), coordinating bursts of AP firing across multiple IHCs (see Section 4 for a detailed description of the mechanism). These studies have also suggested that *ex vivo* IHCs do not fire spontaneous action potentials, which instead are triggered by ATP-mediated mechanisms. Despite ongoing debates regarding the origin of spontaneous action potentials (see Section 5), ATP remains a key regulator of the IHC firing activity. Reducing ATP-induced Ca^2+^ waves in supporting cells, such as through the knockdown of connexin hemichannels, has been shown to alter IHC firing patterns and halt the normal development of the IHCs at pre-hearing stages (Johnson et al., 2017). Moreover, knockout of TMEM16A channels results in reduced electrical activity in the inferior colliculus, increased acoustic sensitivity, and broader frequency tuning, leading to expanded iso-frequency regions (Kersbergen et al., 2022).

#### 3.1.5 Cholinergic efferent feedback

Efferent neurons originating in the brainstem form transient axosomatic inhibitory synaptic contacts with IHCs during immature stages of development (Simmons et al., 1996). Activation of α9α10 nicotinic ACh receptors (nAChRs) in the IHC postsynaptic site leads to a Ca^2+^ influx, which opens closely coupled SK2 channels. This mediates IHC hyperpolarization, thereby reducing cell excitability (Fig. 4) (Glowatzki & Fuchs, 2000; Katz et al., 2004; Marcotti et al., 2004; Roux, Wersinger et al., 2011). As such, the efferent system functions as a negative feedback mechanism that, in principle, can directly modulate the frequency and pattern of spontaneous or evoked action potentials in developing IHCs. However, the exact role of the efferent system on the IHC firing activity *in vivo* remains largely unknown. This uncertainty stems from the fact that cochlear explants lack efferent input, and experimental studies rely on artificial means to mimic its activity. Despite these limitations, several studies have shown that knockdown of α9 nAChRs impairs the maturation of the IHC exocytotic machinery (Johnson et al., 2013). Additionally, in the brainstem, this knockdown disrupts the refinement of the tonotopic map in the medial nucleus of the trapezoid body (MNTB) and affects bilateral coupling in the inferior colliculi (Clause et al., 2014; Wang et al., 2021) (see Fig. 8B and D).

### 3.2 Action potentials in OHCs

Unlike IHCs, much less is known about the origin and modulation of action potentials in OHCs. As discussed in Section 2, OHCs begin acquiring their adult-like biophysical and morphological characteristics by the end of the first postnatal week, approximately a week earlier than IHCs (Fig. 2; Abe et al., 2007; Marcotti & Kros, 1999; Marcotti, 2012; Oliver et al., 1997; Simmons et al., 1996). At this age, the small Ca^2+^ current (Michna et al., 2003) and the presence of the K^+^ current carried by KCNQ4 channels, which are activated at their resting membrane potential (*I*_K,n_: Marcotti & Kros, 1999), prevent OHCs from generating Ca^2+^ action potentials. However, patch-clamp recordings have shown that action potentials can be elicited *ex vivo* in OHCs during the first postnatal week through membrane depolarisation (Marcotti & Kros, 1999; Oliver et al., 1997; Weisz et al., 2012) or elevated extracellular Ca^2+^ concentrations (Beurg et al., 2008). More recent studies using 2-photon imaging or cell-attached voltage clamp have demonstrated that OHCs can also fire spontaneous action potentials when cochlear explants are maintained under physiological Ca^2+^ concentration, but only during the first 3–4 postnatal days (Ceriani et al., 2019; Jeng et al., 2020c; Yang et al., 2023). This narrow time-window of spontaneous firing activity, compared to IHCs, likely results from the earlier onset of functional differentiation in OHCs. Limited information exists regarding the modulation and functional role of spontaneous Ca^2+^ activity in OHCs. A recent study revealed that ATP released by supporting cells binds to ionotropic P2X_3_ receptors on OHCs, increasing their Ca^2+^-induced firing activity (Ceriani et al., 2019). This mechanism was implicated in regulating the maturation of OHCs and their afferent synapses (Ceriani et al., 2019). Furthermore, disruption of the MET channels in OHCs has been shown to impair proper segregation of type II afferent fibres (Sun et al., 2018).

## 4 Calcium signals in developing supporting cells

**Fig. 5:**
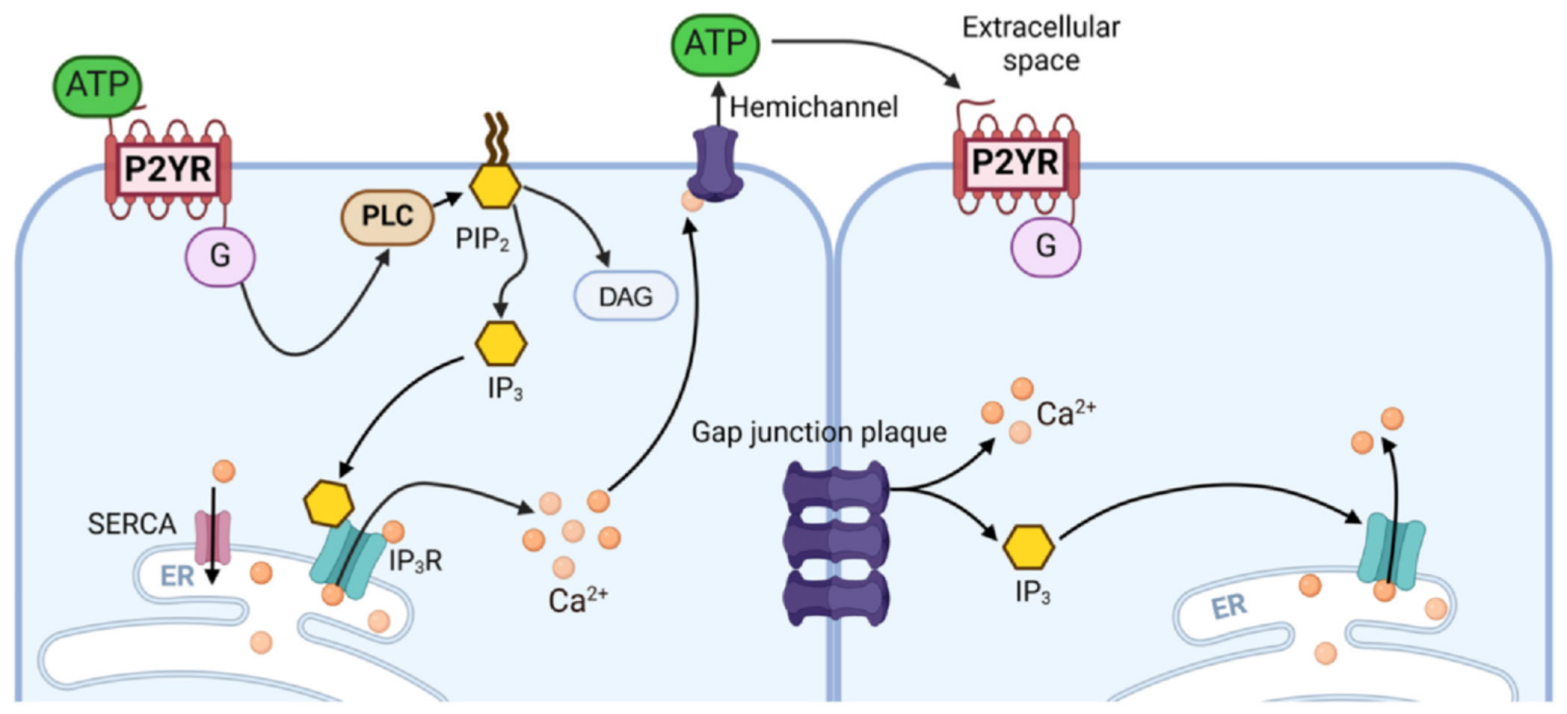
Diagram showing Ca^2+^ signal propagation in supporting cells of the pre-hearing cochlea. Molecular components driving intracellular and intercellular Ca^2+^ signal propagation in supporting cells during cochlear development. ER: Endoplasmic reticulum; PLC: phospholipase C; PIP2; phosphatidylinositol 4, 5 bisphosphate; IP_3_: inositol 1,4,5-trisphosphate; IP_3_R: IP_3_ receptor; DAG: diacylglycerol; G: G-protein; SERCA: sarcoplasmic/endoplasmic reticulum Ca^2+^-ATPase; P2YR: G-coupled metabotropic purinergic receptors. Hemichannels and gap-junction plaques are formed by connexin proteins. Image was created with BioRender.

Experiments have shown that supporting cells in the immature cochlea, in addition to hair cells, exhibit complex Ca^2+^ signalling activity, akin to astrocytes in the CNS (Bazargani & Attwell, 2016). These Ca^2+^ signals, which can propagate as intercellular Ca^2+^ waves throughout the sensory epithelium (Fig. 5), have been implicated in key physiological processes, including cochlear development and damage signalling.

### 4.1 Calcium signalling in supporting cells

Both immature (Ceriani et al., 2016; Gale et al., 2004; Majumder et al., 2010; Piazza et al., 2007; Rodriguez et al., 2012) and mature (Chan & Rouse, 2016; Dulon et al., 1993; Hool et al., 2023; Lagostena & Mammano, 2001; Sirko et al., 2019) cochlear supporting cells respond to the application of adenosine triphosphate (ATP), and related nucleotides, with elevations in their intracellular Ca^2+^concentration.

During pre-hearing stages of development, Ca^2+^ imaging experiments using *ex vivo* cochlear preparations have shown that local application of ATP induces Ca^2+^ oscillations in supporting cells (Ceriani et al., 2016; Gale et al., 2004; Piazza et al., 2007; Rodriguez et al., 2012). Pharmacological studies have indicated that these phenomena follow a canonical G-protein-dependent signalling cascade (Anselmi et al., 2008; Beltramello et al., 2005; Berridge et al., 2000; Piazza et al., 2007): ATP binds to G-protein-coupled P2Y receptors on the supporting cell membrane, leading to the activation of phospholipase C (PLC) enzymes (Fig. 5). Activated PLC hydrolyses phosphatidylinositol 4,5-bisphosphate (PIP_2_) into diacylglycerol (DAG) and inositol 1,4,5-trisphosphate (IP_3_). IP_3_ in turn induces the release of Ca^2+^ from the endoplasmic reticulum into the cytosol through IP_3_ receptors (IP_3_Rs). Calcium oscillations arise from the interplay of Ca^2+^ release and sequestration (*e.g*., through SERCA pumps), and from the bell-shaped Ca^2+^-dependence of the IP_3_R open probability (Bezprozvanny et al., 1991), which cause a biphasic IP_3_R response to intracellular Ca^2+^ (Ceriani et al., 2016; De Young & Keizer, 1992; Li & Rinzel, 1994). Reduction of PIP_2_ synthesis impairs the acquisition of high frequency hearing indicating an important role for this pathway in development (Rodriguez et al., 2012).

The propagation of Ca^2+^ signals across the supporting cells is due to two interdependent mechanisms (Fig. 5). First, gap junctions formed by connexin proteins couple the cytoplasm of neighbouring supporting cells (Forge et al., 2003) allowing them to share the cytoplasmic environment (Zhang et al., 2005). This allows the intercellular diffusion of IP_3_ (Beltramello et al., 2005) propagating the Ca^2+^ signal as an intercellular wave (Anselmi et al., 2008; Beltramello et al., 2005; Majumder et al., 2010; Ortolano et al., 2008). Second, supporting cells can release ATP in the extracellular space either spontaneously or following the extracellular application of ATP onto the hair cells (Anselmi et al., 2008), mechanical stress (Zhao et al., 2005), or damage (Gale et al., 2004; Lahne & Gale, 2008; Lahne & Gale, 2010). Extracellular ATP activates purinergic receptors on neighbouring cells (Anselmi et al., 2008; Zhao et al., 2005), contributing to the intercellular propagation of Ca^2+^ signals (Fig. 5). The spatio-temporal propagation of intra- and intercellular Ca^2+^ signals is further modulated following ATP hydrolysis by ectonucleotidases (Vlajkovic et al., 2002) and mitochondrial buffering (Mann et al., 2009).

The primary candidates for ATP release from supporting cells are connexin hemichannels, which are connexin channels that have no matching hemichannel in the opposing cell membrane (Goodenough & Paul, 2003; Zhao et al., 2005; Anselmi et al., 2008; Majumder et al., 2010). Since hemichannels can open in response to an increase in intracellular Ca^2+^ levels (De Vuyst et al., 2006), it has been proposed that a localised release of ATP by one or a few supporting cells or hair cells can initiate the cascade leading to the long-range propagation of Ca^2+^ waves due to a process termed ATP-induced ATP release (Ceriani et al., 2016). Pannexin channels have also been proposed to underpin the release of ATP in the cochlea (Chen et al., 2015), although other studies have questioned their role (Zorzi et al., 2017; Mazzarda et al., 2020). Indeed, Ca^2+^ signalling due to spontaneous ATP release is unaltered in cochlear preparations from pannexin 1-deficient mice (Zorzi et al., 2017). However, it should be noted that while spontaneous Ca^2+^ signalling was shown to be severely reduced in a *Cx30* mutant (Schütz et al., 2010) and a *Cx30* global knockout (Mazzarda et al., 2020), a recent study has shown that the activity is not affected in the developing cochlea of *Cx26* conditional knockout mice (Kersbergen et al., 2023). Further investigation is needed to completely elucidate the possibly diverse and self-compensating mechanisms responsible for spontaneous ATP release in the developing cochlea.

Ca^2+^ signalling in supporting cells is intimately linked with connexin function in the cochlea. Crucially, mutations in genes encoding connexins that preserve ionic conductance but impair macromolecule permeation through the connexin channel (*e.g*., the V84L CX26 and T5M CX30 mutations: Beltramello et al., 2005; Schütz et al., 2010) have been linked to hearing loss. Moreover, Ca^2+^ signalling in supporting cells can regulate the expression of both Cx26 and Cx30 (Ortolano et al., 2008). Finally, animal models with connexin deletions have shown that connexins are essential for the functional maturation of IHCs, even though the cells themselves do not express connexins (Johnson et al., 2017). Overall, this evidence highlights the importance of Ca^2+^ homeostatic mechanisms in supporting cells for hearing acquisition and normal cochlear function.

### 4.2 Spontaneous calcium waves and their relationship with IHC activity in the prehearing cochlea

During developmental stages (up to the second postnatal week in rodents), the cochlear epithelium comprises two distinct domains: the greater epithelial ridge (GER) lying on its medial aspect and containing the IHCs, and the lesser epithelial ridge (LER), which includes the OHCs, in the lateral aspect (Fig. 1) (Dayaratne et al., 2014). During this period, the GER includes Kölliker’s organ, a transient structure that is prominent during the embryonic and early postnatal stages (Dayaratne et al., 2014) and plays a crucial role in early development and maturation of the auditory system (Hinojosa, 1977).

Supporting cells of Kölliker’s organ spontaneously release ATP into the extracellular space, initiating the signalling cascade that leads to the propagation of Ca^2+^ waves along confined regions of the epithelium (Fig. 5) (Babola et al., 2020; Ceriani et al., 2019; De Faveri et al., 2025; Tritsch & Bergles, 2010; Tritsch et al., 2007; Tritsch et al., 2010). Spontaneous Ca^2+^ waves occur more frequently in the proximity of developing IHCs (de Faveri et al., 2025; Eckrich et al., 2018; Tritsch et al., 2007) and have been shown to trigger the simultaneous depolarization of groups of nearby developing IHCs, modulating their firing frequency and resulting in coordinated bursts of activity along the basilar membrane (Babola et al., 2020; de Faveri et al., 2025; Tritsch & Bergles, 2010; Tritsch et al., 2007; Tritsch et al., 2010). This activity ends after ear canal opening and the onset of hearing (Babola et al., 2021; de Faveri et al., 2025), following the regression of Kölliker’s organ and the downregulation of several key molecules involved in the signalling cascade (Hool et al., 2023; Huang et al., 2010; Wang et al., 2015).

Dissection of the signalling cascade leading to supporting cell-induced modulation of IHC activity (see also Section 3, Fig. 4) has been performed through pharmacological methods and through the study of mutant mice. These studies indicate that ATP released by supporting cells in Kölliker’s organ activates P_2_Y_1_ metabotropic purinergic receptors on neighbouring supporting cells (Babola et al., 2020; Babola et al., 2021). The increased Ca^2+^ in the supporting cells triggers the opening of the calcium-activated chloride channel TMEM16A, which is highly expressed by the supporting cells surrounding the IHCs (Wang et al., 2015), causing efflux of chloride ions. This is accompanied by efflux of water and potassium ions to maintain osmotic balance and electroneutrality (Wang et al., 2015), causing osmotic shrinkage in supporting cells (Tritsch et al., 2007) and depolarisation of neighbouring IHCs (Fig. 4). Finally, the increase in extracellular space due to supporting cell shrinkage quickly dilutes the extracellular potassium concentration, terminating the depolarizing effect on the IHCs and temporally restricting the duration of coordinated burst firing (Babola et al., 2020).

Even though supporting cells of the LER and GER share the same Ca^2+^ signalling cascade (Rodriguez et al., 2012), evidence for spontaneous activity originating in the LER has not been provided. However, Ca^2+^ waves originating in the Kölliker’s organ have been shown to propagate to the LER area in *ex vivo* cochlear explants and organotypic cultures (Ceriani et al., 2019; Majumder et al., 2010) where they trigger depolarization and Ca^2+^ elevation in Deiters’ cells and have been linked to the coordination of spontaneous activity of developing OHCs in mice during early postnatal ages (P0-P4, Ceriani et al., 2019). Further studies will be required to determine whether this type of activity is propagated centrally through type II spiral ganglion neurons *in vivo*.

### 4.3 Other roles for calcium signalling in cochlear non-sensory cells

Besides its role in cochlear development, ATP-dependent Ca^2+^ signalling is also implicated in the sensing of cochlear damage (Gale et al., 2004; Lahne & Gale, 2008; Lahne & Gale, 2010; Piazza et al., 2007). Mechanical damage to hair cells in neonatal cochlear preparations triggers release of ATP from the damaged cells and thus the release of ATP and propagation of Ca^2+^ waves in the supporting cells (Gale et al., 2004; Lahne & Gale, 2010; Piazza et al., 2007). A similar phenomenon can be reproduced by applying a brief puff of ATP to the hair cells (Anselmi et al. 2008; Ceriani et al. 2016). The activation of two distinct intercellular Ca^2+^ waves propagating at different speeds through the supporting-cell and hair-cell regions has been observed (Gale et al., 2004; Lahne & Gale, 2010; Piazza et al., 2007), mediated by both metabotropic (P2Y) purinergic receptors (as described for spontaneous calcium events above) and ionotropic P2X receptors, which drive Ca^2+^ entry from the extracellular space. The propagation of these signals following damage has been linked to the activation of extracellularly regulated kinases 1 and 2 (ERK1/2) (Lahne & Gale, 2008) in cochlear supporting cells, which promote hair-cell death. Although these phenomena have been studied in the neonatal cochlea, the persistence of ATP sensitivity and Ca^2+^ dynamics in the supporting cells of the mature cochlea (Chan & Rouse, 2016; Hool et al., 2023; Park et al., 2024; Sirko et al., 2019) and the role of ATP release during noise exposure (Housley et al., 2013) suggests that they might be important regulatory mechanisms also during normal hearing function.

## 5 Functional integration between cochlear spontaneous calcium signals – an *ex vivo vs in vivo* perspective

Our understanding of how the developing mammalian cochlea functions at the cellular and subcellular level primarily derives from electrophysiological and Ca^2+^-imaging studies performed over the last 30 years. Some of the pioneering work in this area includes studies investigating the development of the basolateral and MET currents (Kennedy et al., 2003; Kros et al., 1992; Kros et al., 1998; Marcotti & Kros, 1999; Marcotti et al., 2003a), the appearance of exocytosis at ribbon synapses (Glowatzki & Fuchs, 2002; Johnson et al., 2005; Moser & Beutner, 2000; Roux et al., 2006) and the potential role of cholinergic efferent synapses in hair cell development (Glowatzki & Fuchs, 2000), and the mechanisms underlying Ca^2+^ waves in supporting cells (Tritsch et al., 2007; Wang et al., 2015). These studies predominantly used organotypic cultures of cochlear explants and, in some cases, implemented artificial means to simulate the *in vivo* environmental conditions characteristic of the mammalian cochlea. Although extremely valuable, these studies have inherent limitations in replicating the cochlea’s complex anatomy, innervation and physiology (Fig. 1). Specifically, these approaches are unable to retain a functional efferent cholinergic input, to mimic the ionic composition of the perilymph and endolymph, and cannot simulate the endocochlear potential, the voltage difference between the scala media and the other cochlear chambers. Consequently, this has limited our ability to understand how the sensory hair cells and supporting cells behave and interact *in vivo*, how the hair cells transmit developmental cues to postsynaptic afferent fibres, and how the cochlear output is regulated centrally by the cholinergic efferent system.

For many years, *in vivo* studies of the mouse cochlea have faced significant technical challenges due to its location deep within the temporal bone and its fluid-filled bony structure. These anatomical constraints limit the access of conventional optical imaging techniques that are essential for investigating individual and multicellular physiological events. However, recent advances have managed to overcome these barriers, providing breakthrough protocols that enable imaging of the different cell types in the developing (De Faveri et al., 2025) and adult (Kim & Ricci, 2022; Kim & Ricci, 2023) mouse cochlea while preserving the *in vivo* physiological conditions. In the following sections, we compare key findings from *in vivo* studies of the developing mouse cochlea (De Faveri et al., 2025) with those previously obtained using an *ex vivo* approach, highlighting how these new approaches enhance our understanding of cochlear development and function.

### 5.1 Calcium signalling in IHCs

**Fig. 6:**
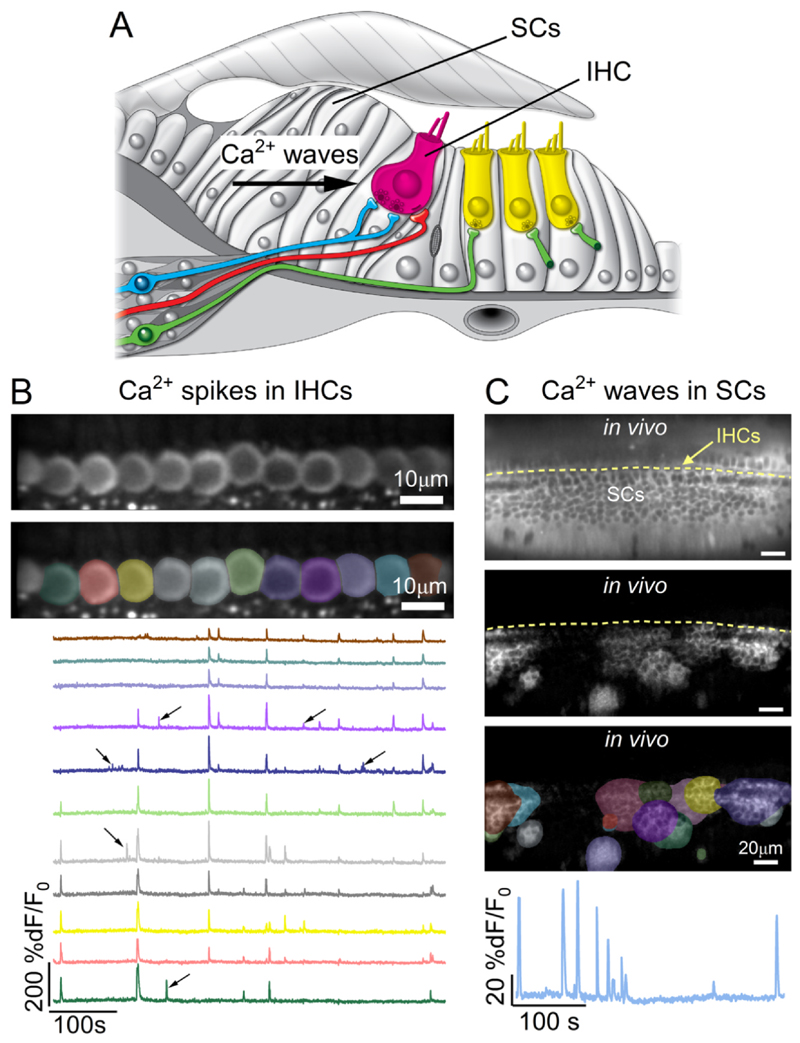
Spontaneous Ca^2+^ signals in supporting cells and IHCs in pre-hearing live mice. (A) Diagram showing a cross-section of an immature organ of Corti. SCs: supporting cells. IHCs: inner hair cells. (B) Average intensity projection of a timelapse recording showing the expression of GCaMP6f in the IHCs from a live pre-hearing *GCaMP6*^*fl/fl*^*Pax2-Cre*^*+/−*^ P6 mouse (top panel) and highlighted regions of interest (ROIs, middle panel), which were used to measure spontaneous Ca^2+^ signals from individual IHCs (bottom panel). Although most Ca^2+^ events are synchronised across adjacent IHCs, most likely due to Ca^2^ waves in nearby supporting cells, uncorrelated Ca^2+^ transients (arrows) indicate intrinsic firing activity (see also De Faveri et al., 2025). (C) Average intensity projection displaying GCaMP6f expression in the supporting cells from a live pre-hearing *GCaMP6*^*fl/fl*^*Pax2-Cre*^*+/−*^ P4 mouse (top panel). The maximal intensity projection showing the Ca^2+^ waves in the supporting cells during a 10-minute recording and related ROI segmentation is shown in the second and third panel, respectively. The bottom panel shows the fluorescence trace from an ROI drawn across the entire supporting cell region. Image from De Faveri et al. (2025).

Although Ca^2+^-dependent action potentials in developing IHCs have been extensively investigated, the cellular mechanisms responsible for their initiation and modulation are still not fully understood. As discussed above (Section 3), some studies have shown that IHCs can spontaneously generate Ca^2+^ spikes at least during the first postnatal week (Beutner & Moser, 2001; Johnson et al., 2011; Kros et al., 1998). By contrast, other studies suggested that these spikes are triggered directly or indirectly by the spontaneous release of ATP from supporting cells (Tritsch et al., 2007; Wang et al., 2015). A recent *in vivo* study has provided new insight, showing that IHCs are able to intrinsically generate spontaneous Ca^2+^ spikes throughout pre-hearing postnatal stages, independent of synchronised activity driven by the Ca^2+^ waves in supporting cells (Fig. 6) (De Faveri et al., 2025). The occurrence of action potentials during the second postnatal week, which are absent in IHCs of *ex vivo* preparations, is likely attributed to the establishment of the endocochlear potential (EP) (Li, Liu, Zhao, & He, 2020) and the distinct ionic compositions of the endolymph and perilymph (Wangemann and Schacht, 1996). The EP, together with the low-Ca^2+^ concentration in the endolymph (Johnson et al., 2012), facilitates a depolarizing MET current through open MET channels (Waguespack et al., 2007; Lelli et al., 2009). This depolarizing current maintains the resting membrane potential of pre-hearing IHCs near the activation threshold of Ca^2+^ channels (Zampini et al., 2010), thereby promoting spontaneous AP activity.

### 5.2 Calcium signalling in supporting cells

Consistent with findings from *ex vivo* studies (see Section 4), supporting cells in the GER of live mice generate spontaneous Ca^2+^ waves throughout pre-hearing stages of cochlear development (Fig. 6). However, the dynamics of these Ca^2+^ waves differ between *in vivo* and *ex vivo* conditions. *In vivo*, Ca^2+^ waves in supporting cells were significantly smaller in size and faster (De Faveri et al., 2025) compared to those observed in cochlear explants (De Faveri et al., 2025; Eckrich et al., 2018; Harrus et al., 2018; Tritsch et al., 2007). Given that ATP is released by supporting cells in response to cochlear damage (Gale et al., 2004; Lahne & Gale, 2008), the large Ca^2+^ waves recorded from cochlear explants, or following the disruption of the *in vivo* physiological conditions (De Faveri et al., 2025), are likely to result from cochlear damage.

### 5.3 Calcium signalling in afferent terminals

Our current understanding of how IHCs transmit information to post-synaptic spiral ganglion neurons (SGNs) primarily stems from electrophysiological patch-clamp recordings performed on individual afferent terminals (reviewed by: Johnson et al., 2019; Moser et al., 2023; Reijntjes & Pyott, 2016), which were pioneered by Glowatzki & Fuchs (2002). However, we still have only a very limited understanding of how the different Ca^2+^ signalling dynamics are faithfully transferred from IHC ribbon synapses to SGN afferent terminals in the developing cochlea. *In vivo* recordings of Ca^2+^ signals reveal that SGN terminals exhibit sporadic Ca^2+^ transients, which are significantly faster than those observed in cochlear explants (De Faveri et al., 2025). Notably, the frequency and amplitude of Ca^2+^ transients recorded *in vivo* are higher in SGN terminals located on the pillar side compared to the modiolar side of the IHCs of pre-hearing mice. Considering that, in the mature cochlea, low-threshold, high spontaneous-rate SGN fibres are predominantly located on the pillar side of IHCs, while high-threshold, low spontaneous-rate fibres are found on the modiolar side (Liberman, 1978; Siebald et al., 2023), these *in vivo* findings suggest that this functional segregation is defined during pre-hearing developmental stages.

### 5.4 Multicellular functional interactions

Although IHCs can intrinsically generate Ca^2+^ spikes *in vivo*, their frequency is significantly increased by spontaneous Ca^2+^ waves originating from supporting cells. These Ca^2+^ waves also play a crucial role in synchronizing Ca^2+^ activity across multiple IHCs, as previously observed in *ex vivo* studies (see Section 4). However, under *in vivo* conditions, Ca^2+^ waves initially synchronize the activity of only a few IHCs, which then spreads longitudinally to recruit additional IHCs (De Faveri et al., 2025). Interestingly, this longitudinal propagation of IHC activity occurs much faster than the Ca^2+^ waves themselves and is independent from Ca^2+^ wave propagation through supporting cells, which was described in cochlear explants (Wang et al., 2015). The mechanism underlying this rapid, longitudinal activation of multiple IHCs remains unclear. One hypothesis could be that K^+^ accumulation in the intercellular space during the repolarisation phase of each AP may lead to the depolarization of the neighbouring IHC.

In live mice, the periodic synchronisation of IHC activity by Ca^2+^ waves has also been shown to increase the proportion of afferent terminals simultaneously activated by a single IHC (De Faveri et al., 2025). The activity among groups of IHCs along the tonotopic axis of the cochlea, which drives synchronous bursts of activity in the developing auditory fibres (Wang et al., 2015; Tritsch et al., 2010), likely contributes to reinforcement and refinement of neuronal projections to discrete areas within the developing auditory pathway (Clause et al., 2014; Müller et al., 2019). Future *in vivo* studies will be essential to identify the role of the efferent cholinergic system in regulating IHC activity during cochlear development.

## 6 Role of spontaneous cochlear activity in maturation of the central auditory pathway

Spontaneous bursts of activity from hair cells are transmitted to SGN (Beutner & Moser, 2001; Glowatzki & Fuchs, 2002) and propagated along the ascending auditory pathway (Babola et al., 2018; Tritsch & Bergles, 2010; Tritsch et al., 2007) where they influence the maturation of central brain structures. This spontaneous activity provides essential input to these central auditory structures before sound-induced activity begins, helping to establish and refine patterns of connectivity and responsiveness, as well as shaping the tonotopic map. While both ascending signals and intrinsically generated spontaneous activity within central auditory structures contribute to circuit maturation, this section focuses specifically on the role of ascending activity generated in the cochlea.

### 6.1 Spiral ganglion neurons require input from inner hair cells for differentiation and maturation

**Fig. 7:**
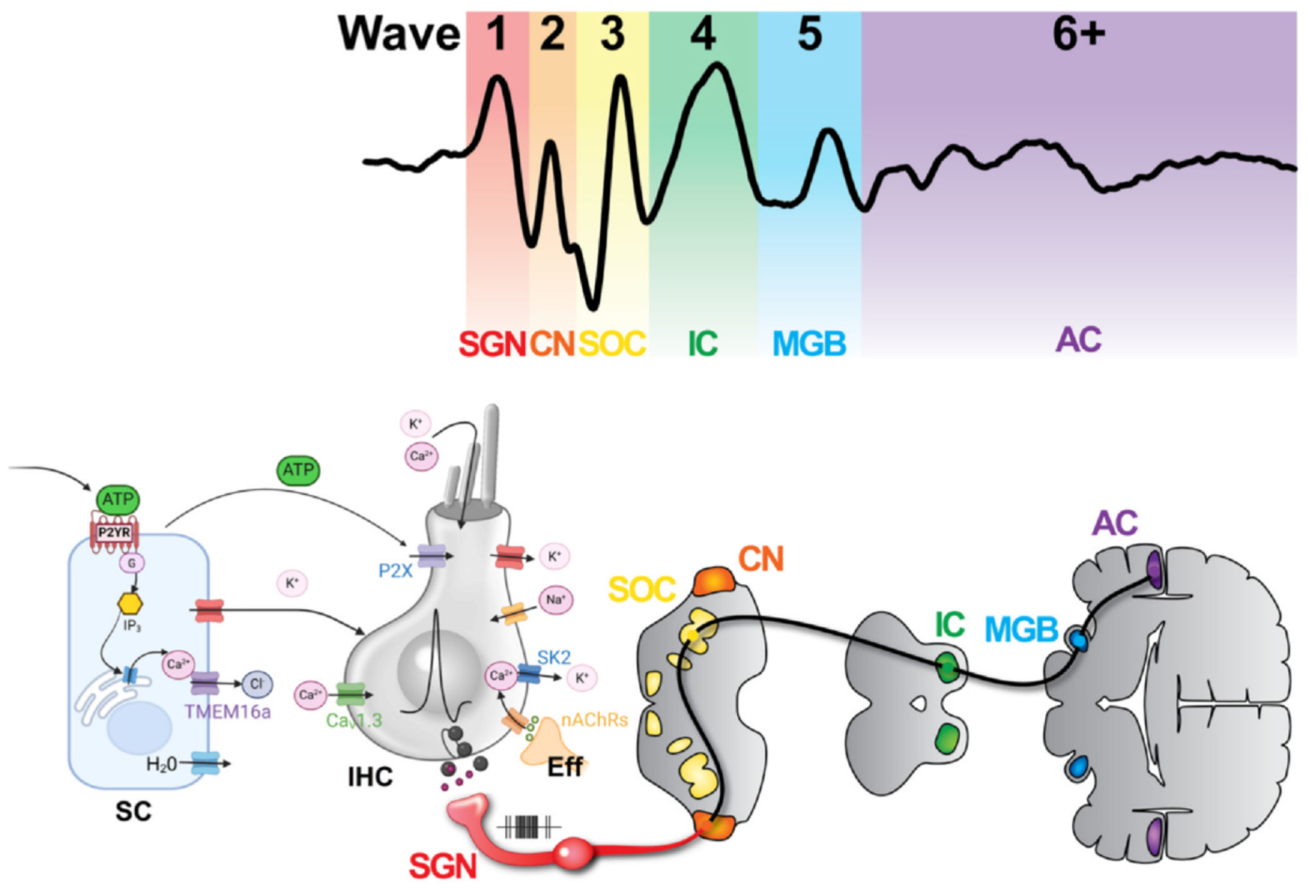
Cochlear spontaneous firing travelling along the auditory pathway. Auditory brainstem responses (ABRs) are used to measure the acoustic signal originating in the cochlear IHCs and travelling along the auditory pathway (top panel). The peaks along the ABR waveform are generated by the electrical activity of neurons located in different parts of the ascending auditory pathway. Wave 1: spiral ganglion neuron (SGN); wave 2: cochlear nucleus (CN); wave 3: superior olivary complex (SOC); wave 4: lateral lemniscus and inferior colliculus (IC). Wave 5 and 6: from the medial geniculate nucleus (MGB) and auditory cortex (AC), respectively. See legend to Fig. 4 for details of components driving/synchronising action potentials in IHCs. The hair cell and supporting cell diagrams were created with BioRender. Image was created with BioRender.

The acoustic signal travelling from the cochlea to the brain can be measured using auditory brainstem responses (ABRs), which provide information about sound processing along the auditory pathway (Fig. 7). The different peaks of the ABR waveform are associated with discrete regions of the ascending pathway from the SGNs up to the auditory cortex (Fig. 7) (Möhrle et al., 2016). The initial stage of sound processing in the ascending auditory pathway is the transfer of acoustic information from the hair cells to the cochlear nucleus (CN) *via* the SGNs, the primary sensory afferent neurons (Fig. 7). Type I SGNs, constituting approximately 90 % of all SGNs, transmit all of the information from IHCs, while type II SGNs (the remaining ~10 % of SGNs) relay input from the OHCs (Ryugo, 1992). Mature type I SGNs each contact a single IHC, with up to 20 SGNs innervating a single IHC, whereas type II SGNs typically contact on average around 7 OHCs (Weisz et al., 2012). Type I afferent synapses are arranged around the base of the IHC from the modiolar to the pillar side, according to their threshold and spontaneous firing rate. High-threshold low-spontaneous rate SGNs are located towards the modiolar side, whereas low-threshold high-spontaneous rate SGNs are positioned towards the pillar side (reviewed by: Johnson et al., 2019). The differentiation, maturation and maintenance of SGNs require the precise timing and coordination of genetic programmes and physiological activity (Delacroix & Malgrange, 2015).

Immature SGNs display three physiologically defined subtypes based on their firing patterns in response to depolarizing current: a single action potential at stimulus onset, a phasic response (with a few spikes at stimulus onset) or tonic firing patterns. During the first postnatal week, these firing patterns change, leaving only fibres with single or phasic responses (Crozier & Davis, 2014; Davis & Crozier, 2015; Prescott et al., 2008). This developmental progression depends on IHC activity since the knockdown of otoferlin, the Ca^2+^ sensor for exocytosis at IHC ribbon synapses (Roux et al., 2006), delays the maturation of SGN biophysical properties (Conrad et al., 2024).

Single-cell RNA sequencing has identified three different genetic expression profiles among SGNs (type 1a, 1b and 1c), which correspond to the spontaneous firing rates and modiolar-pillar synapse location of mature SGNs (Shrestha et al., 2018; Sun et al., 2018). However, these genetic profiles only partially align with physiologically defined subtypes (Siebald et al., 2023). The maturation of these three genetic SGN subtypes relies on IHC activity during pre-hearing developmental stages (Shrestha et al., 2018). It has been shown that type 1a SGNs develop normally in the absence of IHC-driven activity, whereas types 1b and 1c fail to mature properly and instead adopt a type 1a genetic profile (Shrestha et al., 2018). Additionally, disruption of mechano-electrical transduction impairs the initial segregation of immature SGNs into type I and II, and also the separation of type I SGNs into the three different genetic subclasses (Sun et al., 2018).

In OHCs, the absence of SGN firing, resulting from the knockout of the Ca_V_1.3 Ca^2+^ channels, which are essential for generating action potentials and exocytosis, leads to a reduced number of afferent fibres contacting the OHCs (Ceriani et al., 2019). This finding suggests that OHC activity plays a critical role in maturation of type II SGN afferent fibres (Ceriani et al., 2019).

### 6.2 Brain stem structures require cochlear spontaneous input for maturation

Localized firing of groups of adjacent IHCs along the basilar membrane (De Faveri et al., 2025; Tritsch et al., 2010; Tritsch et al. 2010; Wang et al. 2015; Zhang-Hooks et al., 2016) activates SGNs that encode a restricted range of sound frequencies. As outlined above (see Sections 3 and 4), ATP release from inner supporting cells in Kölliker’s organ induces Ca^2+^ waves that synchronise the activation of IHCs tuned to similar sound frequencies in adult mice, suggesting a role in refining tonotopic maps in the central auditory system. This localized, coordinated spontaneous activity in IHCs is transmitted to SGNs *via* specialized ribbon synapses, causing SGNs to exhibit stereotypical bursting activity interspersed with quiescent periods (De Faveri et al., 2025; Jones et al., 2007; Sonntag et al., 2009; Tritsch et al., 2010). This burst firing pattern differs from the continuous, stimulus-driven activity seen in mature SGNs in response to acoustic stimulation (Lippe, 1994; Taberner & Liberman, 2005), suggesting it has a developmental role in shaping the central auditory system. Indeed, recordings in pre-hearing rodents from the medial nucleus of the trapezoid body (MNTB) and the inferior colliculus (IC) have shown similar bursting activity (Sonntag et al., 2009; Tritsch et al., 2010), which is abolished following cochlear ablation (Tritsch et al., 2010).

**Fig. 8:**
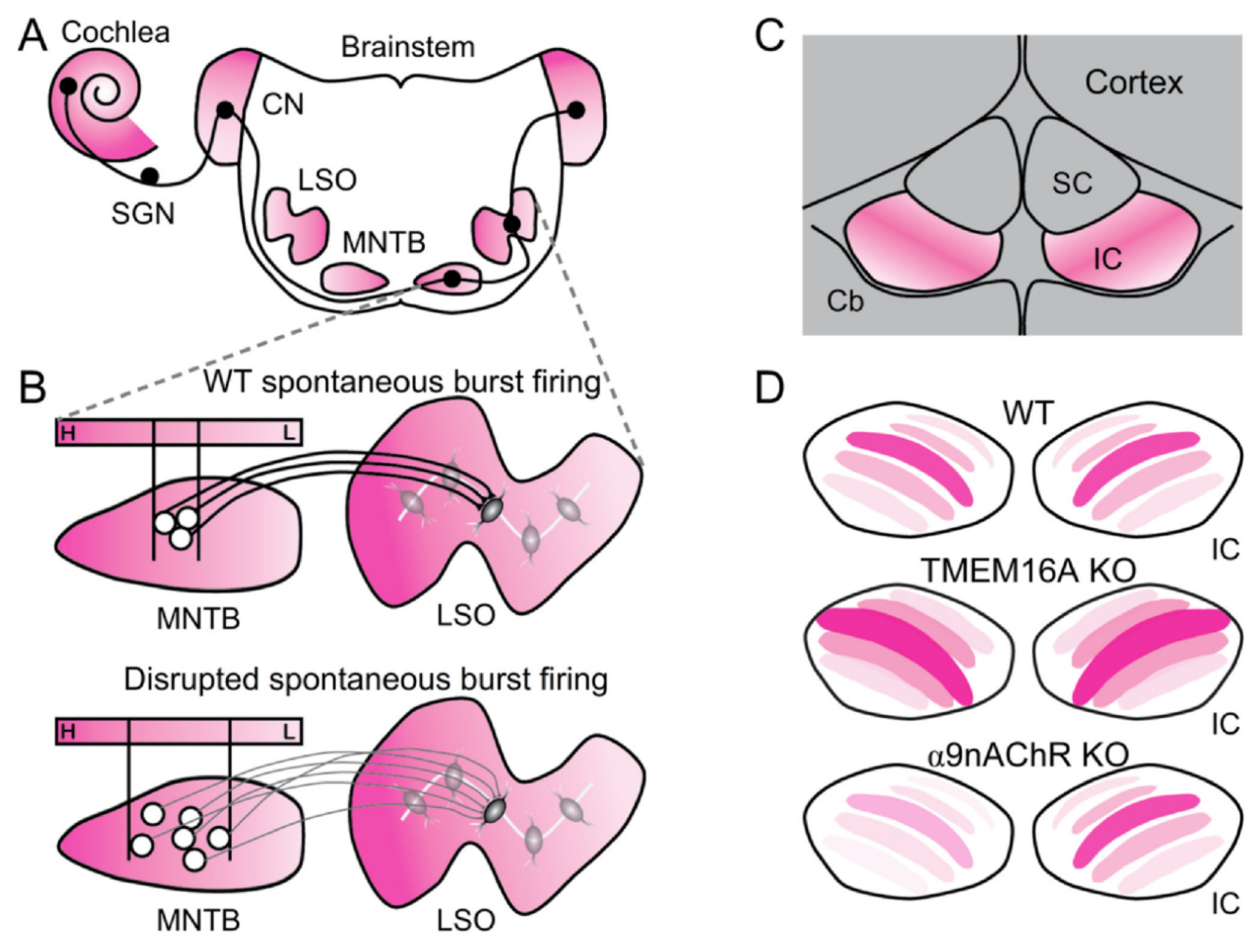
Cochlear spontaneous firing disrupts the central auditory pathway. (A) Diagram outlining the auditory pathway from the cochlea to the brainstem. Spontaneous signals from the cochlea are transmitted to the cochlear nucleus (CN) *via* the spiral ganglion neurons (SGN). The CN projects to the contralateral medial nucleus of the trapezoid body (MNTB) which then projects to the ipsilateral lateral superior olive (LSO). In this and the following panels, the pink shading indicates the characteristic tonotopic organization of the ascending auditory pathway from low (light pink) to high (dark pink) frequency. (B) Diagram illustrating that disrupting the spontaneous burst firing in the cochlea during pre-hearing stages of development through either α9nAChR knockout (Clause et al. 2014) or otoferlin knockout (Müller et al., 2019) leads to impaired strengthening and refinement in the MNTB-LSO circuit and broader input to the LSO from the MNTB.This causes imprecise tonotopy in mice with disrupted cochlear firing compared to wild-type (WT) controls. (C) Diagram showing the location of the inferior colliculus (IC) in the mouse, relative to the superior colliculus (SC) and the cerebellum (Cb). (D) Schematic diagram illustrating the effects of disrupting spontaneous activity in the cochlea on Ca^2+^ transients in the IC. In wild-type (WT) mice Ca^2+^transients in the IC spread along the isofrequency bands of the tonotopic axis correlated across both colliculi. In TMEM16a knockout mice the colliculi had compressed tonotopic maps (Kersbergen et al., 2022). In α9nAChR knockout mice, the activity becomes less symmetrically correlated across the colliculi (Babola et al., 2021). *B Adapted from* Müller et al. (2019). Figure adapted from Kersbergen and Bergles (2024).

Genetic knockout of otoferlin, which prevents the transfer of spontaneous activity from the IHCs to the SGNs, leads to a consequent loss of spontaneous firing in the medial nucleus of the trapezoid body (MNTB) (Müller et al., 2019). This loss of activity impairs MNTB-lateral superior olive (MNTB-LSO) synaptic refinement and strengthening, reducing the precision of MNTB-LSO topographic connections (Fig. 8A and B) (Müller et al., 2019). Similarly, TMEM16A-conditional knockout mice, which likely have disrupted and unsynchronised IHC firing activity *in vivo*, show severely diminished burst firing in MNTB neurons before hearing onset, and reduced frequency selectivity shortly after hearing onset (Maul et al., 2022; Kersbergen & Bergles, 2024). These findings underscore the role of cochlear IHC burst firing in refining auditory connections to form the tonotopic axis (Maul et al., 2022). Naturalistic bursting patterns imposed on MNTB-LSO circuit in brain slices induce robust long-term synaptic potentiation, highlighting the importance of the burst firing pattern in tonotopic map refinement (Bach & Kandler, 2020). Further evidence comes from studies on mice with IHCs lacking α9nAChRs, which show normal spontaneous action potential activity but with altered frequency and timing (Johnson et al., 2011; Vetter et al., 1999). This change in spike pattern temporal structure leads to impaired synaptic strengthening, over-retention of MNTB-LSO connections, disrupted functional MNTB-LSO map refinement before hearing onset, and deficits in sound localization (Clause et al., 2014; Clause et al., 2017).

Calcium imaging in awake transgenic mice expressing genetically encoded Ca^2+^ indicators has revealed transient, highly correlated bouts of activity in IC neurons and auditory cortex (AC) along iso-frequency contours of the tonotopic axis prior to hearing onset (Babola et al., 2018). This coordinated activity has been attributed to cochlear-driven spontaneous firing, as bilateral cochlear ablation or pharmacological blockade of P2Y1 purinergic receptors (disrupting IHC burst firing), abolished the coordinated activity in the IC. Additionally, knockout of α9-nAChRs, essential for efferent feedback to the cochlea, reduces symmetrical correlated bilateral activation across the left and right colliculi (Fig. 8C and D), whereas a gain-of-function mutant increases this correlation (Babola et al., 2021).

TMEM16A knockout in the mouse cochlea has been shown to suppress IC neuron burst firing pre-hearing, resulting in broader tuning curves, higher neuronal gain, and compressed tonotopic maps in both the IC and AC (Fig. 8C and D) (Kersbergen et al., 2022). This indicates that spontaneous activity in the IC is required for the refinement of tone responses and connectivity in the tonotopic axis. Despite the AC tonotopic map compression, AC neurons maintain normal response properties, possibly due to alternative sources of spontaneous activity and post-hearing refinement (Babola et al., 2018; Molnár et al., 2020). Overall, spontaneous activity in the brainstem is required for CNS tonotopic organisation and the refinement of brainstem neuron response properties.

### 6.3 Auditory cortex

The role of peripheral spontaneous activity in shaping the development of the auditory cortex is less well understood. The thalamus and cortico-thalamic loops are established before peripheral inputs reach the cortex, including topographically organized connections within the subplate neurons (Molnár et al., 2020), which are part of the first postmitotic cortical neuronal layer. These neurons respond to peripheral auditory stimulation before responses are seen in the primary recipient layer of the auditory cortex (Wess et al., 2017), potentially explaining the presence of tonotopic maps in individuals with congenital deafness (Striem-Amit et al., 2016). In the absence of peripheral activity, the somatosensory thalamus retains an immature state, with each division resembling one of a ‘higher-order’ (Pouchelon et al., 2014). This suggests that higher-order thalamic-cortical connections may represent the default pattern, implying that peripheral input refines the medial MGB, the primary auditory thalamic subfield (Molnár et al., 2020), and cortical tonotopic maps (Babola et al., 2018).

## 7 Revisiting development in the ageing cochlea

Auditory function declines with age to varying degrees, with hearing thresholds initially increasing at high frequencies and then, more gradually, increasing at lower frequencies (reviewed by: Elliott et al., 2022). The rate of this decline is influenced by genetic predisposition, lifetime exposure to excessive sound levels, or to otototoxic drugs such as aminoglycoside antibiotics (Bowl & Dawson, 2019; Liberman, 2017). While age-related hearing loss is primarily associated with loss of OHCs, recent research highlights significant changes in and around the IHCs (Jeng et al. 2020a; Jeng et al., 2020b; Jeng, et al., 2021; Lauer et al., 2012; Zachary & Fuchs, 2015;).

### 7.1 Efferent reinnervation of IHC due to trauma or ageing

Age-related changes in IHCs were first suggested by observations of cochlear innervation by the efferent system. In the mature cochlea, MOC efferent fibres directly innervate OHCs, whereas LOC efferent fibres indirectly influence the output of IHCs by synapsing onto their type I afferent nerve fibres (Fig. 1D). Following acoustic trauma or overexposure to AMPA (which mimics a toxically high concentration of the IHC neurotransmitter glutamate), type I nerve fibres endings on guinea-pig IHCs swelled and then disappeared. Within hours of this happening, efferent nerve fibres began to contact the IHCs directly (Ruel et al., 2007), as seen during cochlear development. This direct efferent reinnervation of IHCs has also been observed in ageing mice (8–11 months), associated with loss of type I afferent nerve fibres, OHC loss, and increased ABR thresholds (Lauer et al., 2012). However, recent evidence indicates that in adult IHCs, efferent innervation can occur even in the presence of afferent fibres (Corns et al., 2018; O’Connor et al., 2024). Functionally, these newly formed efferent synapses inhibited IHCs by activating SK channels (Jeng et al., 2021; Zachary & Fuchs, 2015), similar to their role during cochlear development (Marcotti et al., 2004).

### 7.2 Loss of MET current leads to regression of IHCs to an immature state

Studies on mice with mutations in various hair-bundle proteins have demonstrated that an absence of MET current significantly impacts the maturation of IHCs and OHCs (see also Section 3). For instance, constitutive mutations in the MET channel pore protein TMC1 inhibit the normal development of hair cells, preventing the expression of *I*_*K*,*f*_ and *I*_*K*,*n*_ (see Fig. 2) Consequently, IHCs retain the ability to fire action potentials and exhibit immature neurotransmitter release properties (Marcotti et al., 2006). Similar developmental impairments were observed in myosin VI mutant mice, where the hair cells lack MET currents (Roux et al. 2009), as well as in mice with mutations in the Usher-syndrome proteins myosin VIIa, harmonin and protocadherin 15 (IHCs and OHCs: Roberts, 2013; IHCs: Corns et al., 2018). This suggests that the MET current is not only required for IHC maturation, but also for maintaining their mature phenotype. Compelling evidence in support of this hypothesis comes from experiments where myosin VIIa was conditionally knocked out. In these mice, the MET current functioned normally during development but gradually diminished over time, becoming undetectable by P30 (Underhill et al., 2025). Initially, the IHCs acquired the mature potassium currents (*I*_*K*,*f*_ and *I*_*K*,*n*_, see Fig. 2), developed mature presynaptic activity, and normal hearing as measured by ABRs (Corns et al., 2018). However, from P20 onwards, *I*_*K*,*f*_ and *I*_*K*,*n*_ declined, disappearing entirely by P60, and the conditional myosin VIIa knockout mice were deaf. During the same time window, IHCs became smaller, re-expressed immature IHC currents (*I*_*K1*_ and *SK2*), responded to ACh application with inhibitory *SK2* currents, and became re-innervated by efferent fibres.

Further experiments using cochlear cultures from mature gerbils highlighted the crucial role of the resting MET current (which is approximately 10% of the maximal MET current active without hair-bundle stimulation or sound) in preserving the IHC mature physiological characteristics (Corns et al., 2018). In culture, where the hair bundles are not stimulated by sound, *I*_*K*,*f*_ progressively disappeared over five days in culture. Interestingly, reducing extracellular Ca^2+^ to mimic *in vivo* endolymphatic levels, which increases the size of the IHC resting MET current, slowed the decay of *I*_*K*,*f*_, unless the MET current was pharmacologically blocked.

The findings of Corns et al., (2018) show that mature IHCs regress to an early developmental state within weeks following the loss of their MET current, to a stage where they fire spontaneous action potentials. This regression can result from mutations in MET-associated hair bundle proteins (as observed by Corns et al., 2018), noise-induced damage, or ageing. Although this process is much slower than the reversible stereocilia shortening that occurs within hours when the Ca^2+^ influx *via* MET channels is reduced (*e.g*. by MET channel blockers; Vélez-Ortega et al., 2017), it likely stems from a deficit in Ca^2+^ influx through the MET channels (see also Velez-Ortega and Frolenkov **Chapter 2** this volume for further details). This Ca^2+^ influx may regulate gene expression in the cells by diffusing into the cytoplasm (Beurg et al., 2010).

### 7.3 Regression of IHCs and their innervation in the ageing cochlea

A study on the biophysical properties of ageing mice (over one year old; Jeng et al., 2021) revealed changes similar to those reported by Corns et al., (2018) following the gradual reduction of myosin VIIa in hair cells. However, the extent of these changes varied depending on the mouse strain’s susceptibility to age-related hearing loss (Jeng et al., 2021). In C57BL strains with the cadherin 23 mutation (*Cdh23*^*ahl*^) which leads to early-onset age-related hearing loss, IHCs became smaller, resembling pre-hearing IHCs, had reduced MET currents and showed a significant decrease in *I*_*K*,*f*_, although *I*_*K*,*n*_ was maintained. In mice in which the inherent cadherin 23 mutation was repaired, the progression of age-related hearing loss, as measured by ABR recordings, was slower but still present, and the reductions in *I*_*K*,*f*_ and cell size were less pronounced. In the mouse strain most resistant to age-related hearing loss (C3H), IHCs displayed minimal change, with only a slight reduction in size with age. Direct efferent reinnervation also varied according to the hearing loss phenotype. However, even in C3H mice, some reinnervation and reappearance of post-synaptic inhibitory SK2 currents in response to ACh were observed (Jeng et al., 2021). This efferent reinnervation likely involved LOC fibres, which typically innervate afferent nerve terminals below IHCs, as opposed to MOC fibres that transiently contact IHCs during cochlear development *en route* to the OHCs (Simmons et al., 1996). This reinnervation of IHCs appears to replace afferent synapses that are lost during age-related hearing loss, particularly those formed by the high-threshold, low-spontaneous rate fibres on the modiolar side of the IHCs (Jeng et al., 2021).

Interestingly, OHCs also exhibit spontaneous action potentials early in development (Jeng et al., 2020a) and fail to express *I*_*K*,*n*_ in the absence of the MET current, similar to IHCs. This suggests that OHCs may undergo regression in a similar way to IHCs when MET current input diminishes or ceases. However, OHCs do acquire their characteristic electromotility even without the MET current (Roberts, 2013) or action potential activity (Jeng et al. 2020a). In the ageing cochlea, the primary change observed in OHCs is a reduction in cell size, accompanied by a proportional decreases in *I*_*K*,*n*_, electromotility and sparse afferent innervation (Jeng et al., 2020b). These changes occur regardless of the mouse strain’s susceptibility to early age-related hearing loss. Conversely, reductions in MET current size and loss of efferent synaptic connections are associated with early age-related hearing loss (Jeng et al., 2021) due to the cadherin 23 mutation (*Cdh23*^*ahl*^).

## 8 Final remarks and conclusions

Over the last 20 years or so, a detailed understanding of the functional maturation of the cochlear sensory epithelium has emerged. Precisely choreographed interactions of calcium signals and action potentials between different cell types in the epithelium, as well as between the cochlea and the rest of the auditory pathway, enable the auditory system to mature despite the absence of sound input.

Recent research has revealed the dynamic properties of spontaneous activity within the *in vivo* cochlea, shedding light on key interactions between the spontaneous activity of IHCs and supporting cells (De Faveri et al., 2025). *In vivo* approaches offer a novel experimental paradigm that can address unresolved questions regarding cochlear function, not only during pre-hearing stages but also in adult mice. For instance, our understanding of the role of the inhibitory MOC efferent system in modulating the spiking activity of developing IHCs remains limited, as these connections are severed in cochlear explant preparations. Additionally, it is unclear how IHC firing patterns control glutamate release at the different ribbon synapses around their basolateral membrane, and how this is encoded by type I SGN terminals *in vivo*. Previous studies have identified the second postnatal week as a critical period during which IHC action potentials regulate the morphological and physiological maturation of the IHCs themselves (Johnson et al., 2013; Carlton et al., 2024). However, the intracellular signals regulating the underlying mechanisms are still unknown. While OHCs exhibit spontaneous Ca^2+^ activity within a narrow postnatal window in mice, it remains uncertain whether, like IHCs, this activity contributes to the refinement of type II SGNs and associated brainstem circuits. Addressing these questions will enhance our understanding of how the cochlea influences the refinement of the developing auditory system. Future research will benefit from continued advances in genetically-encoded indicators, which, when combined with the *in vivo* approach developed for both the pre-hearing (De Faveri et al., 2025) and adult cochlea (Kim & Ricci, 2023), will facilitate investigations of auditory processing down to the single-synapse level. This integration will help to bridge current knowledge of single-cell physiology with systems-level understanding in the cochlea.

Moreover, future studies are needed to clarify the relationship between the signalling pathways that drive cochlear development and the morphological and physiological changes that occur in the ageing cochlea, particularly as mechano-electrical transduction in the IHCs begins to fail and immature features reappear. It is currently unknown whether aged IHCs regain the ability to generate spontaneous Ca^2+^ action potentials. However, if they do, and by receiving reinnervation from efferent system, aged IHCs may establish bidirectional communication with the brain, independent of sound input. This raises important questions for hearing preservation and restoration: Could this efferent rewiring help maintain the integrity of the remaining afferent nerve fibres, despite a reduced or absent MET current? This would be important as intact afferent nerve fibres are essential for cochlear implants to effectively bypass IHCs that cannot be stimulated by sound (Marzella & Clark, 2009). Conversely, could this rewiring be detrimental, potentially contributing to tinnitus by signalling non-existent inputs to the brain? Another intriguing possibility is whether the ‘immature’ IHCs of the aged cochlea can be stimulated to regain mature functionality through regenerative or gene therapies (see also Walters, Cox and Stone **Chapter 7** this volume for further details) A recent study demonstrated that AAV-mediated gene therapy, administered shortly after the normal onset of hearing, could restore myosin VIIa function in conditional myosin VIIa knockout mice (O’Connor et al., 2024). Remarkably, the regression of the mature IHCs to an earlier developmental state – evidenced by the downregulation of *I*_*K*,*f*_ and their efferent reinnervation – was reversable in 1–2 month old mice, with partial restoration of hearing function. These findings offer promising potential for applying similar approaches to restore hearing in the ageing cochlea. Ultimately, the signalling circuits established during cochlear development are surprisingly relevant for understanding and potentially reversing age-related hearing loss. Insights gained from developmental studies may therefore pave the way for novel therapeutic strategies to combat hearing loss in old age.
